# Reductions in the Prevalence and Incidence of Geohelminth Infections following a City-wide Sanitation Program in a Brazilian Urban Centre

**DOI:** 10.1371/journal.pntd.0000588

**Published:** 2010-02-02

**Authors:** Luciene Maura Mascarini-Serra, Carlos A. Telles, Matildes S. Prado, Sheila Alvim Mattos, Agostino Strina, Neuza M. Alcantara-Neves, Mauricio L. Barreto

**Affiliations:** 1 Departamento de Parasitologia, Instituto de Biociências, Estadual University of Julio Mesquita Filho (UNESP), Botucatu, São Paulo, Brazil; 2 Instituto de Saúde Coletiva, Federal University of Bahia, Salvador, Bahia, Brazil; 3 Departamento de Ciências Exatas, Estadual University of Feira de Santana, Bahia, Brazil; 4 Instituto de Ciências da Saúde, Federal University of Bahia, Salvador, Bahia, Brazil; George Washington University Medical Center, United States of America

## Abstract

**Objective:**

In the city of Salvador, a large urban centre in Northeast Brazil, a city-wide sanitation intervention started in 1997, aimed at improving the sewerage coverage of households from 26% to 80%. Our aim was to study the impact of the intervention on the prevalence and incidence of geohelminths in the school-aged population.

**Methods:**

The study comprised two comparable cohorts: the first assembled in 1997, before the intervention, and the second assembled in 2003, after the intervention. Both were sampled from 24 sentinel areas chosen to represent the different environmental conditions throughout the city. Copro-parasitological examinations were carried out on every individual from both cohorts, at baseline and nine months later. Demographic, socio-economic, and environmental data were collected using semi-structured questionnaires and environmental surveys. A hierarchical modelling approach fitting a sequence of Poisson multivariate linear models was undertaken to test the effect of the intervention variables on the prevalence and incidence rate ratios.

**Findings:**

729 and 890 children aged 7–14 years (mean = 10.4 y, SD = 0.05 y) were analysed over the first and the second cohorts, respectively. The adjusted reductions of the prevalence and incidence rates at the second in relation to the first cohort were 27% and 34%, 25% and 32%, 33% and 26%, and 82% and 42% for geohelminths overall, *Ascaris lumbricoides, Trichuris trichiura*, and hookworm, respectively. Hierarchical modelling showed that a major part of each of these reductions was explained by the intervention.

**Conclusion:**

Our results show that a city-wide sanitation program may reduce significantly the prevalence and incidence of geohelminths.

## Author Summary

In the city of Salvador, a large urban centre in Northeast Brazil, a city-wide sanitation intervention started in 1997, aiming to improve the sewerage coverage of households from 26% to 80%. Our aim was to study the impact of the intervention on the prevalence and incidence of geohelminths in the school-aged population. The longitudinal study comprised two cohorts: from the beginning of 1997 to 1998, where data was collected before the intervention, and at the end of 2003 to 2004, after the intervention. Copro-parasitological examinations were carried out on every individual from both cohorts, at the start and nine months later. Demographic, socio-economic, and environmental data were collected using semi-structured questionnaires. The variables utilized to demonstrate the effects of intervention, when utilized together in a multivariate model, accounted for a 100% observed reduction in the prevalence ratio (PR) and incidence ratio (IR). As well as proving that the variables associated with the effect of the program intervention were mediators in this reduction, the reduction in the PR and IR between these periods demonstrated that modifications to the urban environment, particularly those associated with sanitary sewage systems, affected the health of the population, significantly reducing the prevalence of geohelminths.

## Introduction

Soil-transmitted helminths, or geohelminths, form one of the most important groups of infectious agents and are the cause of serious global health problems; more than a billion people are currently infected by at least one species of this group of pathogens [1]. At a global level, the most important geohelminths are roundworms (*Ascaris lumbricoides*), whipworms (*Trichuris trichiura*), and hookworms (*Necator americanus* and *Ancylostoma duodenale*); it is estimated that, respectively, these parasites have infected 1.2 billion, 800 million, and 740 million people [1]. In Brazil alone it is estimated that 41.7 million people are infected with *A. lumbricoides*, 18.9 million with *T. trichiura*, and 32.3 million with hookworms [2].

Geohelminths are more frequently found among children living in conditions of poor sanitation, and their impact on morbidity and mortality is more severe in malnourished populations [3]. Most studies suggest that approximately 70% of the worm population is hosted by 15% of the human host population. These few, heavily infected individuals are at a higher risk of disease and are also the prime source of environmental contamination [4]. Inadequate hygiene, poor health care systems and facilities , and social indifference make this situation worse. However, geohelminth control is often neglected, even in highly worm-infested countries.

Geohelminths usually coinfect the host. Especially children living in deprived environments from less-developed areas may be chronically infected with more than one worm [5],[6]. Such children have increased risks of malnutrition, stunted growth, mental retardation, and cognitive and learning deficiencies [1].

Large-scale environmental sanitation programs are complex, making interventions directly aimed at the transmission of geohelminths a challenge [7]. These interventions directly affect the transmission of several diseases in both the public and private domains [8]. Several factors should be considered for such an intervention to be successful. Amongst these are public investment in sewerage networks which must be matched by individual households' willingness to invest in a toilet and connect it to this network [7].

There are no studies of the health effects of sanitation intervention programs in large cities of developing countries. An extensive program of environmental sanitation was conducted in the Brazilian city of Salvador, Bahia aimed at expanding the city's sanitation network from 26% to 80%. A marked reduction in the rate of childhood diarrhoea has already been reported since the sanitation intervention program [7]. In this study, our aim was to report the impact of this environmental sanitation program on the prevalence and incidence of geohelminths among school aged 7–14 years.

## Methodology

The study was conducted in the city of Salvador, in the state of Bahia, Brazil. Bahia has an estimated population of 2.8 million inhabitants as of 2007 [9]. Before the intervention approximately 26% of the households was connected to the city sewage system, and it was thought that the remainder used alternative methods of sanitation (such as septic tanks) or simply disposed of their waste on the street. The program started in the mid-1990s with the objective to increase the sewerage coverage to 80% of households.

The evaluation of the impact of the program, known as *Bahia Azul (Blue Bay)*, on the occurrence of intestinal parasites in children and teenagers of school age (7–14 years) took place in two phases: the first, from 1997 to 1998, involved the collection of pre-intervention data, and at the second, from 2003 to 2004, involved the collection of post-intervention data by which time over 60% of residences were connected to the sewage network. In each case the studies were of a longitudinal design with two cohorts in 1997–1998 and 2003–2004.

The procedure for choosing the areas studied (sentinel areas) has been described in detail elsewhere [7],[10]. These areas were selected to represent the poor, unsewered part of the city, which, before introduction of the sanitation programme in 1997 (the intervention), represented about 75% of the population. Each sentinel area represented about 600 households. A sample of households with children and adolescents aged 7–14 years was randomly selected from a census of each sentinel area, and only one eligible child per household was randomly chosen to be enrolled in the investigation. The sample was stratified proportional to the number of school-aged children present in each of the sentinel areas.

In both cohorts the populations used were in the same age range (7–14 years at baseline) and the same methods were used for data collection. Demographic, socio-economic, environmental, and sanitary information was collected by appropriately trained field-workers using semi-structured questionnaires given to parents or guardians of the schoolchildren. Environmental inquiries conducted in 1997 and 2004 allowed for the definition of contextual variables in each sentinel area [11]. Samples of faeces were collected twice from each studied individual from both cohorts at baseline and approximately nine months later. The copro-parasitological methods of spontaneous sedimentation [12] were used to identify eggs, protozoan cysts, and the Kato Katz method [13] for quantification of helminth eggs. After each examination, the children were treated for any parasitic infection.

Prevalence and incidence rates (PR and IR) were calculated for each geohelminth. To calculate the incidence in each cohort, those shown to be positive at the second examination but negative at the first were divided by the individuals negative at the first examination and re-examined at the second examination. Prevalence and incidence rate ratios were constructed by dividing the rate (of prevalence or incidence) of the second cohort (2003–4) by the corresponding rate in the first cohort (1997–8). The analyses comprised univariate and multivariate analyses. A hierarchical modelling [14] approach involved fitting a sequence of hierarchical Poisson regression multivariable log-linear models to test the effect of the intervention variables on the prevalence and incidence rate ratios. The PR and IR were obtained from the hierarchical model as the Poisson regression coefficient comparing post- versus pre-intervention periods, with standard errors calculated from a robust covariance matrix.

**Figure 1:**
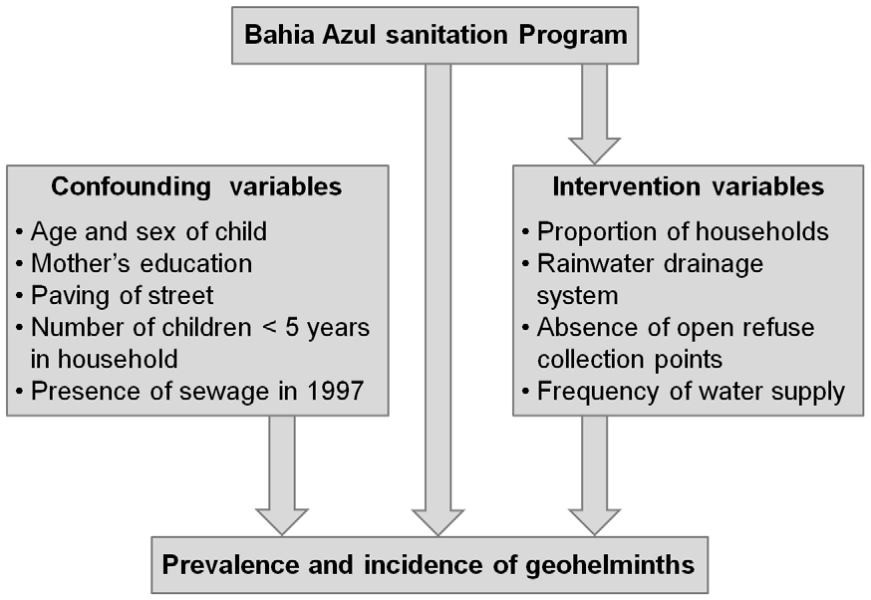
Conceptual model to investigate the effect of the city-wide sanitation intervention (*Bahia Azul* program) in prevalence and incidence of geohelminths.

A conceptual model (Figure 1) presents the confounding variables and the variables related to the intervention (mediating factors). It is worth noting that the *Bahia Azul* program, besides its main intervention—sanitation—had complementary actions on water supply, garbage collection, and rainwater drainage. Based on this model, variables were selected and measured in the pre- and post-intervention studies. These variables were: the proportion of households connected to the *Bahia Azul* sewage system, the proportion of households with a regular water supply, the proportion of households connected to the drainage system, and the percentage of households without points of sewage. All four variables presented significant changes in between the two studies (Table 1). Confounding variables were included in the regression equations in stages to measure the effect of the intervention on geohelminths. Confounding variables not associated with the program that were included were: sex, age of the child, maternal educational level, street paving, number of children <5 years of age in household, and the presence of some type of sewage system in 1997. Cluster adjustment to account for the 24 sentinel areas was also added to the analysis. Attributable fraction (AF) was also estimated; this is the proportion of reduction that can be attributed to the intervention variables. The data were analysed using the STATA (ver.9.0) statistical program.

**Table 1:** The frequency distribution of intervention and confounding related variables used in the hierarchical multivariate models, before and after the city-wide sanitation intervention.

Variables	Categories	Before Intervention (N = 729)			After Intervention (N = 890)		
		n	%	95% CI * (%)	n	%	95% CI * (%)
**INTERVENTION**							
**Presence of drainage system of rainwater**	1° tercile (≤0.05)	276	37.9	34.3–41.5	239	26.8	23.9–29.8
	2° tercile (>0.05 and ≤0.21)	211	30.3	27.0–33.8	319	35.8	32.6–39.0
	3° tercile (>0.21)	232	31.8	28.4–35.3	332	37.3	34.1–40.5
**Absence of open refuse collection points**	1° tercile (≤0.80)	211	28.9	25.7–32.4	226	25.4	22.5–28.3
	2° tercile (>0.80 and ≤0.89)	273	37.4	33.9–41.0	327	36.7	33.5–40.0
	3° tercile (>0.89)	245	33.6	30.1–37.1	337	37.8	34.7–41.1
**Frequency of households with regular water supply**	1° tercile (≤0.44)	380	52.1	48.4–55.8	144	16.2	13.8–18.7
	2° tercile (>0.44 and ≤0.60)	156	21.4	18.5–24.5	360	40.4	37.2–43.7
	3° tercile (>0.60)	193	26.5	23.3–29.8	386	43.5	40.1–46.7
**Proportion of households connected to the ** ***Bahia Azul*** ** sewage system**	≤25% of houses in area	729	100	—	100	11.2	9.2–13.5
	>25%, ≤50% of houses	0	0	—	160	18.0	15.5–20.6
	>50%, ≤75% of houses	0	0	—	322	36.2	33.0–39.4
	>75% of houses	0	0	—	308	34.6	31-4–37.8
**CONFOUNDING**							
**Age**	7–10 years	337	46.2	42.5–50.0	520	58.4	55.1–61.7
	11–14 years	392	53.7	50.1–57.4	370	41.6	38.3–44.9
**Sex**	Female	354	48.6	44.9–52.2	408	45.8	42.5–49.2
	Male	375	51.4	47.7–55.1	482	54.2	50.8–57.5
**Mother's education**	≤4 years	208	28.9	25.3–32.9	202	22.7	20.0–25.6
	>5 and ≤11 years	361	50.1	45.8–53.2	517	58.9	54.8–61.3
	>12 years	151	20.9	17.8–23.8	171	19.2	16.7–21.9
**Paving of street**	Paved	314	43.1	39.4–46.7	302	33.9	30.8–37.1
	Unpaved	415	56.9	53.2–60.5	588	66.1	62.8–69.2
**Number of children <5 years in household**	<2	342	46.9	43.2–50.6	742	83.4	80.7–85.7
	>2	387	53.9	49.4–57.0	148	16.6	14.2–19.2
**Presence of sewage in 1997**	1° tercile (≤0.37)	247	33.9	30.4–37.4	340	38.2	35.0–41.5
	2° tercile (>0.37 and ≤0.66	253	34.7	31.2–38.3	278	31.2	28.0–34.4
	3° tercile ( >0.66)	229	31.4	28.0–35.0	272	30.6	27.5–33.7

* CI = exact binomial 95%.

After each parasitological examination, the children found positive for the investigated geohelminths received appropriate treatment or were directed to a health service. Written informed consent to participate in the study was obtained from the children's parents or guardians, according to the study protocol approved by the Research Ethics Committee of the Federal University of Bahia (UFBA).

## Results

A total 1,619 children were studied, 729 in the pre-intervention (1997–8) and 890 in the post-intervention cohort (2003–4; “baseline”). Respectively 390 and 356 tested positive for some geohelminth at baseline. The overall prevalence of all three studied geohelminths was 53.5% in the pre-intervention group and 40.0% in the post-intervention group (p<0.001) (Table 2). Regarding the prevalence of each geohelminth, in 1997, 42.9% were positive for *T. trichiura*, 33.1% for *A. lumbricoides*, and 9.9% for, hookworms. In 2003, prevalences were 28.8% for *T. trichiura*, 25.5% *A. lumbricoides*, and 1.7% hookworms. All parasite infections decreased statistically significantly between the two periods. The overall incidence for all three geohelminths in the pre-intervention period (1997–8) was 36.0% and in the post-intervention period (2003–4), 25.7%, a statistically significant difference. The parasites with the highest incidence both in 1997–8 and in 2003–4 were *A. lumbricoides* and *T. trichiura*, although only *A. lumbricoides* showed a statistically significant reduction after the intervention.

**Table 2:** Prevalence and incidence of geohelminths in the studied population, before and after the city-wide sanitation intervention.

Geohelminths	Prevalence					Incidence						
	1997 (n = 729)		2003 (n = 890)		p*	1997–1998			2003–2004			p*
	pos	%	pos	%		n**	pos#	%	n**	pos#	%	
***Ascaris lumbricoides***	241	33.1	227	25.5	0.001	488	100	20.5	663	101	15.2	0.02
***Trichuris trichiura***	313	42.9	256	28.8	0.001	416	46	11.1	634	56	8.8	0.23
**Hookworms**	72	9.9	15	1.7	0.001	657	15	2.3	875	12	1.4	0.18
**Any geohelminth**	390	53.5	356	40.0	0.001	400	144	36.0	584	150	25.7	0.001

* Difference between periods = test χ^2^.

** n = negative in first exam.

# pos = positive in the second exam.

The crude prevalence rate ratio (PR) observed for the three geohelminths together was 0.74 (95% confidence interval [CI], 0.57–0.89) and the prevalence rate ratio adjusted for confounders (age, sex, street paving, number of children <5 years in household, maternal educational level, and presence of sewage system in 1997) was 0.73 (95% CI, 0.66–0.81). Therefore, a reduction of 27% was seen in the overall prevalence of geohelminths following the sanitation program (Table 3).

**Table 3:** Prevalence rate ratio (PR) of geohelminths before and after the city-wide sanitation intervention, adjusted for confounding and intervention variables.

Prevalence Ratio (PR)	Any Geohelminth (95% CI)	AF*%	*Ascaris lumbricoides* (95% CI)	AF*%	*Trichuris trichiura* (95% CI)	AF* %	Hookworms (95% CI)	AF*%
PR (crude)	0.74 (0.57–0.89)	—	0.77 (0.64–0.92)	—	0.67 (0.56–0.79)	—	0.17 (0.09–0.29)	—
PR adjusted^A^	0.73 (0.66–0.81)	—	0.75 (0.63–0.87)	—	0.67 (0.58–0.79)	—	0.18 (0.09–0.33)	—
PR adjusted^B^	0.74 (0.66–0.83)	3.7	0.76 (0.63–0.91)	4.0	0.68 (0.58–0.75)	3.0	0.17 (0.09–0.29)	−1.2
PR adjusted^C^	0.72 (0.65–0.80)	−3.7	0.74 (0.62–0.87)	−4.0	0.66 (0.58–0.75)	−3.0	0.17 (0.09–0.31)	−1.2
PR adjusted^D^	0.77 (0.68–0.88)	14.8	0.81 (0.66–0.98)	24.0	0.71 (0.59–0.86)	12.1	0.20 (0.11–0.37)	2.4
PR adjusted^E^	0.96 (0.89–1.04)	85.2	1.14 (0.97–1.36)	100	0.83 (0.74–0.91)	48.5	0.23 (0.09–0.56)	6.1
PR adjusted^F^	1.24 (0.98–1.58)	100	1.72 (1.26–2.34)	100	1.06 (0.72–1.58)	100	0.37 (0.12–1.09)	23.2

*: AF = Proportion of reduction attributable fraction of intervention variable.

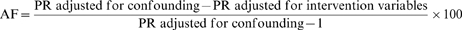

A Ratio adjusted for confounding: age and sex of child, paving of street, number of children <5 years in household, mother's education and presence of sewage in 1997.

B Ratio adjusted for variable of intervention = presence of drainage system of rainwater and for confounding ^A^.

C Ratio adjusted for variable of intervention = absence of open refuse collection points and for confounding ^A^.

D Ratio adjusted for variable of intervention = frequency of water supply and for confounding ^A^.

E Ratio adjusted for variable of intervention = proportion of households connected to the *Bahia Azul* sewage system and confounding ^A^.

F Ratio adjusted for all variables of intervention ^B+C+D+E^ and for confounding ^A^.

The mediating variables of the intervention were introduced in stages (Table 3). The presence of a drainage system for rainwater and the absence of open refuse collection points had a small impact on the size of AF, showing variations of 3.7% and −3.7%, respectively. When the frequency of water supply variable was added, the AF was 14.8%; however, the greatest proportion of variation occurred with the introduction of the variable for the proportion of household connected to the *Bahia Azul* sewage system, with an AF of 85.2%. When all the variables, including both the confounders and the intervention variables, were added to the model, the decrease in the overall geohelminth prevalence was fully explained.

Considering each geohelminth species, the adjusted prevalence rate ratio for *A. lumbricoides*, *T. trichiura*, and hookworms showed reductions of 25%, 33%, and 82%, respectively. When we analyse the intervention variables and the proportion of reduction attributable to the intervention variable (AF), we can demonstrate that for *A. lumbricoides* the variable for frequency of water supply accounted for 24% of the observed reduction, and the proportion of households connected to the *Bahia Azul* sewage system variable accounted for 100% of the observed reduction. For *T. trichiura* the variable for frequency of water supply accounted for 12.1% of the observed reduction, and the proportion of household connections to the *Bahia Azul* sewage system accounted for 48.5% of the observed reduction. When all variables were combined, 100% of the reduction of *T. trichiura* was explained. For hookworms, the effect of the sewage was the lowest observed, given that the proportion of household connections to the *Bahia Azul* sewage system accounted for 6.1% of AF, and that all combined intervention variables achieved an AF of 23.2% (Table 3).

For the three geohelminths, the crude incidence rate ratio (IR) between the periods analysed (2003–4 and 1997–8) was 0.71 (95% CI = 0.57–0.89) and the IR adjusted by the confounders was 0.66 (95% CI = 0.55–0.79) with a 34% reduction between the periods (Table 4).

**Table 4:** Incidence rate ratio (IR) of geohelminths before and after the city-wide sanitation intervention, adjusted for confounding and intervention variables.

Incidence Ratio (IR)	Any Geohelminths (95% CI)	AF*%	*Ascaris lumbricoides* (95% CI)	AF* %	*Trichuris trichiura* (95% CI)	AF* %	Hookworms (95% CI)	AF* %
IR (crude)	0.71 (0.57–0.89)	—	0.74 (0.56–0.98)	—	0.80 (0.54–1.17)	—	0.60 (0.28–1.28)	—
IR adjusted^A^	0.66 (0.55–0.79)	—	0.68 (0.51–0.90)	—	0.74 (0.54–1.02)	—	0.58 (0.35–0.93)	—
IR adjusted^B^	0.68 (0.55–0.82)	5.9	0.70 (0.51–0.94)	6.2	0.73 (0.52–1.02)	−3.8	0.59 (0.34–1.0)	2.4
IR adjusted^C^	0.64 (0.54–0.76)	−5.9	0.67 (0.50–0.88)	−3.1	0.75 (0.56–1.0)	3.8	0.51 (0.30–0.88)	−16.6
IR adjusted^D^	0.71 (0.56–0.89)	14.7	0.71 (0.49–1.03)	9.4	0.82 (0.58–1.15)	30.8	0.64 (0.34–1.18)	14.3
IR adjusted^E^	0.79 (0.66–0.94)	38.2	0.82 (0.60–1.10)	44.0	0.85 (0.19–1.57)	42.3	1.31 (0.84–2.04)	100
IR adjusted^F^	1.00 (0.59–1.66)	100	1.09 (0.65–1.81)	100	0.95 (0.19–2.16)	80.8	—	—

*: AF = Proportion of reduction attributable fraction of intervention variable.

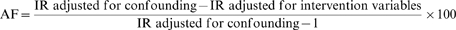

A Ratio adjusted for confounding: age and sex of child, paving of street, number of children <5 years in household, mother's education and presence of sewage in 1997.

B Ratio adjusted for variable of intervention = presence of drainage system of rainwater and for confounding ^A^.

C Ratio adjusted for variable of intervention = absence of open refuse collection points and for confounding ^A^.

D Ratio adjusted for variable of intervention = frequency of water supply and for confounding ^A^.

E Ratio adjusted for variable of intervention = proportion of households connected to the *Bahia Azul* sewage system and for confounding ^A^.

F Ratio adjusted for all variables of intervention ^B+C+D+E^ and for confounding ^A^.

When the same model with mediating variables was applied to the incidence data (Table 4), the presence of rainwater drainage systems accounted for 5.9% of the reduction, frequency of water supply accounted for 14.7%, the proportion of household connections to the *Bahia Azul* sewage system accounted for 38.2%, and the combination of all intervention variables accounted for 100% of the reduction. The same model was also applied to each geohelminth species using incidence data which gave an adjusted IR for *A. lumbricoides* of 0.68 (95% CI = 0.51–0.90) signifying a reduction of 32% between the periods. The observed reduction in the variable presence of rainwater drainage accounted for 6.2%, the variable frequency of water supply accounted for 9.4%, the proportion of household connections to the *Bahia Azul* sewage system for 44%, and all the variables of the model accounted for 100% of the reduction. For *T. trichiura* a statistical difference in the crude or in the adjusted was not shown, however, in combination the AF observed for the intervention variables was 80.8%.

For hookworms an adjusted IR of 0.58 (95% CI = 0.35–0.93), meaning a reduction of 42%, was observed; when the AF of the intervention variables was analysed, the AF of the variable frequency of water supply was 14.3% and the variable proportion of household connections to the *Bahia Azul* sewage system accounted for 100% of the observed reduction. Because of the small number of cases the model with all intervention variables was not estimated (occurrence of overlap).

## Discussion

Our results show that a city-wide sanitation program may significantly reduce the prevalence and incidence of geohelminths infections. After controlling for potential confounders, the observed reductions in the prevalence and incidence adjusted rates at the post-intervention cohort in relation to the pre-intervention one were 27% and 34% for all geohelminths, 25% and 32% for *A. lumbricoides*, 33% and 26% for *T. trichiura*, and 82% and 42% for hookworms. With the hierarchical modelling, it was observed that a major part of each of these reductions was explained by the sanitation intervention.

High prevalences of *A. lumbricoides* and *T. trichiura* have been found in the Salvador population over several decades, showing that geohelminths are highly endemic in this city. Faria et al. [15], in the 1960s, reported prevalence rates in schoolchildren from public schools of 76.5%, 97.8%, and 36.2% for *A. lumbricoides*, *T. trichiura*, and hookworms, respectively. Moraes et al. [16], two decades later, in a population of the same age range in locations lacking any sanitary infrastructure, found prevalence rates for the same helminth species of 66.4%, 87.8%, and 25.2%. Similarly high prevalence of geohelminths has been found in children resident in periurban areas within other developing countries, all of which exhibit deficiencies in environmental sanitation [17]–[19].

In 1997, our results of the pre-intervention period showed an overall prevalence for the three studied geohelminths of 53.5%, with 33.1% for *A. lumbricoides*, 42.9% for *T. trichiura*, and 9,9% for hookworms. A few years later, when the *Bahia Azul* sanitation intervention had taken place, the overall prevalence as well as the prevalence of each individual geohelminth showed significant reductions. In contrast to previous studies published in the literature, the present study's use of similar methodologies and the potential confounders to compare the results in the pre- and post-intervention periods permitted the inference that there were effective reductions in the prevalence and incidence of geohelminths in the course of the sanitation intervention. In order to analyse the extent to which the intervention contributed to these reductions, we used a hierarchical modelling strategy and AFs were estimated. For the three geohelminths together, 85.2% of the reduction was due to the rise in the proportion of households connected to the new sewage system that occurred in the period between the two surveys. It was also responsible for 100%, 48.5%, and 6.1% of the observed reduction in the prevalence rates of *A. lumbricoides*, *T. trichiura*, and hookworms, respectively. When this variable was combined with other variables related with the intervention (the presence of drainage water system, absence of open refuse collection points, frequency of water supply) and confounders, the observed reductions in prevalence rates were fully explained for geohelminths overall, *A. lumbricoides*, and *T. trichiura*, but not for hookworms (only 23% of the prevalence reduction was explained) (model F; see legends of Tables 3 and 4). In contrast to *A. lumbricoides* and *T. trichiura*, the mechanism of transmission of hookworms is centred in the peri-domestic environment. The efficiency of hookworm transmission increases when the infective stage (L3 larva) find a humid environment with high temperatures, substantial rainfall, and sandy soil. Other unmeasured environmental factors [3],[20] and other interventions [21] could have interfered with the drastic reduction of prevalence of these parasites adequately estimate this specific type of transmission.

The scarcity of data on geohelminth incidence makes difficult any comparison with the incidence rates found in pre- and post-intervention cohorts in Salvador. It is known that the level of prevalence of geohelminths is the cumulative effect of the level of incidence over time. Besides the environmental factors, prevalence could also be affected by non-environmental factors such as chemotherapy, widely used in their treatment and control [5],[22],[23].

However, the incidence, in contrast to the prevalence, is much more dependent on environmental factors. Consequently, the effect of an environmental intervention is best measured by its effect on the incidence. Between pre- and post-intervention periods there were important reductions in the incidence rates for geohelminths overall and for each specific geohelminth studied. In our models an important part of this reduction could be attributed to the rise in the proportion of households connected to the new sewage system. This was fully explained by this variable, and the other three variables related with the intervention (model F).

Our results are a clear demonstration that changes in the urban environment, particularly those associated with sewage sanitation, affect the population's health by reducing the prevalence and incidence rates of geohelminths infections. It has already been shown that the implementation of this sanitation program was followed by a reduction of 22% in the prevalence of diarrhoea in pre-school children, and this reduction was fully explained by the intervention [7].

An adequate sewage system is a sustainable strategy of disease control, bringing various benefits to the health of the population [24]. Public investments in sanitation are essential to protect individuals from open air effluents or effluents running in the streets and to control geohelminths and other sanitation-related infectious diseases [7],[25].

The importance of basic sanitation is incontestable. According to UNICEF and WHO [26], basic sanitation can prevent 1.5 billion children from dying of diarrhoea-related diseases and protect the health of millions of people. Even today, throughout the world, 2.6 billion people (including one billion children) do not have access to sanitation, meaning that only 62% of the world's population has access to sanitation infrastructure that allows for the adequate disposal of human excrement.

## Supporting Information

Checklist S1 STROBE checklist.
(0.09 MB DOC)
